# Students' Learned Helplessness and Teachers' Care in EFL Classrooms

**DOI:** 10.3389/fpsyg.2021.806587

**Published:** 2021-12-15

**Authors:** Hua He

**Affiliations:** School of Foreign Languages, Zhengzhou Normal University, Zhengzhou, China

**Keywords:** EFL classrooms, students' learned helplessness, educational self-efficacy, EFL (English as a foreign language) learning, teachers' care

## Abstract

The construct of learned helplessness, as one of the noticeable emotional issues in education, has been investigated and approved its prominent place in education for its stimulus on students' accomplishment, enthusiasm, and commitment in learning. Moreover, the role of teachers as the most crucial issues in the learning viewpoint is at the center of attention since they help learners to be more motivated and provide a supportive context by caring. So, the current review of literature tries to emphasize teachers' care and its effects on decreasing learned helplessness in EFL classrooms. The review of this study underscores the important role of the teachers' care and support in learners' improvement of learned helplessness that can be alleviated in this way. Afterward, some suggestions are offered to illuminate the exercise of teachers, learners, teacher trainers, and syllabus designers.

## Introduction

Foreign language learners are all aware of the significance of attaining good English abilities, but some strike as not being motivated in the educational cycle. Their absence of interest in their participation is an example of discouragement which will subsequently lead to depression and demotivation (Hsu, 2011). Mostly, being scared of failing, being irresponsible, and escaping from completing tasks could result in failures (Sorrenti et al., 2004). Teachers have a hard time identifying the reasons for learners' failure or absence of perseverance in assignments, which affects learners' learned helplessness (LH) (Gordon and Gordon, 2006). Failure is bound to happen in any educational cycle. Nevertheless, what puts an end to education is not failure but the students' response to it, which happens to also mold their education (Daggol, 2018). Attributions are the grounds for individuals' achievements or failures when performing a specific assignment and regular attributes in a scholastic environment include endeavor, innate capability or intellectual capacity, difficult assignments, educators' support, and individual help (Horner and Gaither, 2004). Learners do not have real courage to solve issues and deal with difficulties, and they lack persistence and they tackle most assignments with a deconstructive behavior, an absence of inspiration, and inherent emotions of failure as well as incompetence (Wurm, 2021). These learners would rather ignore doing assignments or carry out simpler ones; therefore, they would not face disappointments that are regarded as a reflection of their innate absence of abilities. Moreover, when learners lack the capability of changing their reaction to difficult circumstances like coping with frustration and repetitive failures, and an essential inspiration or self-determination, they are then mentally evasive and possess LH (Eldowah and Alnajashi, 2017). They are incapable of altering or provoking the strategy fulfilled after their failure. Therefore, they ascribe no worth to their dedication, as they believe that they have no control over their educational cycle and their scholastic achievement (Dickhäuser et al., 2011). Students are at a danger of contracting LH when they face an academic failure cycle and they become less aware of their skills (Pasta et al., 2013). Consequently, such learners demonstrate a deconstructive attitude toward academic assignments, cease to be involved, and develop destructive feelings like frustration and stress (Filippello et al., 2018). Learned helplessness is an outcome of recurrent perception that the result of a certain stressful circumstance has nothing to do with the practice of a person in that circumstance (Peterson, 2010). The LH mindset can ultimately have serious deconstructive effects on a person in which extreme levels of LH create self-abandonment in learners and the mental discomfort resulting from LH leads to feeling lost, mentally void, depressed in students, and ultimately develop false views on values and life (Wu and Tu, 2019). It is not solely their attitudes that are self-destructive; rather the ones with them experience similar feelings when it comes to getting the job done and confronting hardships. When learners graduate, these practices and attitudes do not suddenly disappear, rather they affect their life after graduation, and it is passed on to adulthood, work-life, social life, and psychological well-being (Wurm, 2021). Out of the elements that have an important function in this field, Carson et al. (2002) connected LPs with educators' practices because the learners who were not supervised by the educator were essentially helpless. while strict teaching behaviors like mental control can contribute to anxiety and poor psychological well-being, the self-determination theory (SDT) proposed that care from others like parents and educators is crucial for learners' achievement (Ryan and Deci, 2017). Moreover, student-educator interactions are significant in different domains that can greatly affect a learner's development and growth and as a matter of fact, self-esteem is more likely to occur when learners feel that they are actively supported by their class educators (Jones and Hensley, 2012; Xie and Derakhshan, 2021). Indeed, the educator plays a role in a classroom's success in language education and their efficacy can affect learners' accomplishments or failures in education as Raufelder et al. (2013) discovered that learners at school who were self-aware, experienced less LH because they had a better connection with their educators. Learners' conceptions of educator's care illustrate significant aspects of learner–educators associations (Wentzel, 2016). Teachers' care inspires learner-related competencies like commitment, self-assurance, well-being, and success (Derakhshan et al., 2019; Sun, 2021). Moreover, the students who are cared for by their educators are exposed to a low level of LH as a result educators should have healthy relationships that care about their students in diverse circumstances which hinder their LH (Wang and Eccles, 2012).

Additionally, LH impedes educational success (Filippello et al., 2020), so, on the one hand, ignoring or not paying attention to LH in foreign language education contribute to worse outcomes since such learners affect others and influence identical practices (Sucu and Bulut, 2019) and on the other hand, adequate attention has not been paid to LH in the scholastic setting and this is likely due to it not being identified by educators as a state that could bring about grave issues in learners, like depression (Filippello et al., 2018). Remaining in this LH condition can leave the person less inspired to carry out new practices for the sake of dealing with deconstructive occurrences, being overwhelmed by a feeling of frustration that is closely associated with depression and stress (Filippello et al., 2018). The most difficult and distressing aspect of educators' experience was caring about learners who slowly lose their interest in English education and became LH. So by evaluating associated research on learned helplessness, founded on the scholar's information, numerous investigations solely emphasized the influences of educational self-efficacy on learned helplessness as well as the influence of educators' anticipations on learned helplessness in general education (Putwain and Symes, 2014; Wu and Tu, 2019) and, the role of teachers' care has not been much investigated in EFL context so far. Therefore, the present review aimed to fill this gap by inspecting the function of teachers' care on students' LH in EFL classrooms.

## Review of Literature

### Learned Helplessness

Learned helplessness can be explained as an intricate layout of flawed attributions that have been actualized as fundamental to a cognitive-behavioral hypothesis of dissatisfaction and that can also assist with clarifying some behavioral issues like a weak academic achievement (Schleider et al., 2014). In addition, LH is a mental condition defined by an inner, fixed, and international style of attribution for failures; an exterior, unsteady, and particular attribution style for success; absence of self-confidence in one's skills and intellect; interpretation prejudice of occurrences; and deconstructive prediction of achievements (Filippello et al., 2020) and it refers to an impediment learners face in language education as Hall et al. (2008) regard it as a mental state that causes people to think they have lost control of an adverse circumstance, that their actions are pointless, and that they are defenseless. Also, LH has been described by Saxena and Shah (2008) as a mental state in which animals or humans cannot control the ineffectiveness of their endeavors and they maintained that this type of belief is followed by becoming passive even when exposed to detrimental or deconstructive circumstances. In the school context, LH is a cycle oriented toward dominance on the one hand and helplessness on the other hand; consequently, learned helpless people demonstrate less perseverance in the assignment as well as a lower will to succeed when the assignment seems difficult (Odabasi, 2013).

### Teachers' Care

As stated by Mayseless (2015), caring is a feeling, an attachment, and a behavioral demonstration that surrounds all human beings and can be explicated as an emotion, an inspiration, and/or a practice, mirroring worries about the emotions and demands of others. For a safe and viable educator-learner association, educators' care is a significant element that can add to a learner's growth (Lavy and Naama-Ghanayim, 2020; Wang and Guan, 2020). Caring and emotionally comforting associations with educators are especially significant for undervalued outnumbered learners and English students that is one of the mechanisms of safe, current educator-learner rapport, which can be effective in learners' progress. Educators' caring is an intricate concept that involves hearing out and being interested in what learners would like to relay, considering their emotions, developing trust, and assisting them with attaining their optimum capacity (Noddings, 2012).

## Implications and Future Directions

Learned helplessness is a construct created mainly in psychology and is intertwined with numerous other notions, all of which are demonstrated to learners through teaching. With the help of the literature review, it becomes clear that there are many ways in which teachers can help their learners reach their highest potential. So this review can add to the literature by confirming the key function of the educator in caring about LH students' accomplishment. Although students learn specific practices and attitudes during their education, there are methods educators can apply to assist learners with reducing LH and creating resilience. Educators need to help learners with observing their endeavors' effects on their achievement, rather than their talents or incompetence. The current review underlines the nature of educators' care and their emotional comfort like their kindness or reception to learners' demands can keep students away from developing deconstructive relational results. Through teacher care, social ties are created between teachers and learners in which the learners feel appreciated and encouraged leading to students' motivation and persistence that consequently decrease their LH.

Along with the prominence of academic improvements in the EFL setting, educators should devote more time to constructing caring behavior that increases their self-efficacy, promotes their language education, and lessens or even prevents their LH. Indeed, educators are advised to recognize helpless learners and take them for granted and be careful to avoid comparing them to one another or avoid creating settings that foster competition. Moreover, some approaches are endorsed to be utilized in the classroom that would motivate helpless learners to challenge themselves. In this way, the content developed by the syllabus designers should be motivating enough which trigger their interests.

Failure is an expected issue in the process of learning; nevertheless, it is not failure that stops education, then again it is student's response to failure that forms his/her learning that leads learners to gradually lose attentiveness in learning and develop their LH and to lessen the degree of LH in English education, learners must be helped with developing suitable goals when it comes to learning English and this happens with educators' guidance. When learners perceive that their educator pays attention to them, they develop greater self-assurance and a constructive educator-learner association is created at school, which leads to a lower degree of LH at school. Learners' feeling that their educator cares for them is probable to promote the level of learners' trust and constructive feeling toward the learners and trust was interconnected to start a positive relationship with teachers and caring has been deemed a basis for an encouraging their relationship that encompasses a significant construction which permits educators to accomplish the learners' desires with empathy and warmth (Mayseless, 2015). With the help of adequate levels of educator's care, learners are capable of becoming more autonomous and positive concerning their scholastics and this shows that deconstructive attributions for achievement and failure can be substituted to decrease LH. A great degree of emotional comfort from educators kept learners with high degrees of scholastic disengagement.

Educator training must consider the impacts of caring educators on learners and create plans that strengthen educator caring capabilities and aware educators of its potential influences and possible antecedents. To strengthen their responsiveness to learners' feelings and to react positively to their emotional demands, educators must be educated as it appears that developing a constructive educator-learner is a precondition for enhancing scholastic achievement. Furthermore, direct interference is necessary because neglecting learners' helpless practices, in the long run, can render them unreactive or late reactive. Also, the participation of learners in a self-inspired manner must be enhanced with the help of controlling their objectives and anticipations. As LH is an important concept, more research must be conducted from various dimensions, and more interference ought to be made to decrease the LH in English education. Studies should be led to observe the optimum methodologies for overcoming LH that is regarded as extreme and expand over numerous settings.

## Author Contributions

The author confirms being the sole contributor of this work and has approved it for publication.

## Funding

This study was supported by Humanities and Social Science Project (Youth Project) administered by the Education Department of Henan Province in 2021-Research on the Training of University Normal Students Based on British CELTA Concept (No.: 2021-ZZJH-480).

## Conflict of Interest

The author declares that the research was conducted in the absence of any commercial or financial relationships that could be construed as a potential conflict of interest.

## Publisher's Note

All claims expressed in this article are solely those of the authors and do not necessarily represent those of their affiliated organizations, or those of the publisher, the editors and the reviewers. Any product that may be evaluated in this article, or claim that may be made by its manufacturer, is not guaranteed or endorsed by the publisher.
